# Making a Fortune

**Published:** 1864-10

**Authors:** 


### MAKING A FORTUNE.

Some men can make money any where; they will get rich on a rock; others strive laboriously all their lives, succeed at nothing and die poor. The former have promptness, energy, system and forethought. Some get rich by trickery, meanness and habitual deceptions; others by straightforward dealing and a generous remuneration and humane care of all whom they employ. These principles have found a striking illustration in the history of one of the handsomest retail stores on Broadway. At No.505 a few days ago, we saw a hundred happy looking girls at work; not in a damp musty basement; not in a cobwebbed and stifling hot attic; not in some unsunned crib with bare brick walls and rough joists or rafters overhead, but in an elegant apartment on the ground floor; large, light and cheery; quietness, tidiness every where prevailing. Being well paid and well cared for, every girl is anxious to maintain her place; and as the best means of doing so, does her work in the most thorough manner; to make assurance doubly sure, that lynxeyed embodiment of all that is tasteful and befitting, Mrs. E. Wintle, examines the work in detail as it progresses as well as after its completion, lest a thing might be allowed to "pass" sometimes when it ought not to, because of the "trouble" of undoing so much. Passing by mantillas, cloaks, dresses, and a thousand and one other things worn by ladies and children, there was one little item about gentlemen's underclothing which was characteristic of the whole establishment. If a stranger or citizen wants a shirt on an emergency all ready, or to be made, it is uniformly presented to him, elsewhere, as thick as a board with starch, with the express object of concealing the slaziness of the material. It glistens like the frozen snow and vies with it in whiteness; you can hear its rattle and rustle, on handling, a hundred yards away, but on the very first washing its worthlessness is apparent at a glance; the garment will scarcely bear half a dozen washings. Now all this contemptible skinning is done away with, at Scott & Baldwin's establishment; their goods are "soft finished;" are scarcely starched at all; nothing is done to conceal the quality of the article; you can see for yourself what it is at the first glance and are never disappointed in its wear. Our readers will not be disappointed to learn of the success of such a house, and it is a satisfactory confirmation of the truth of the time honored legend (although some, in these days of shoddy, have almost begun to question it), that "HONESTY IS THE BEST POLICY."

